# Irritable bowel syndrome-like symptoms are associated with increased healthcare utilization in patients with inflammatory bowel disease

**DOI:** 10.3389/fgstr.2026.1924176

**Published:** 2026-09-22

**Authors:** Sophie Montgomery, Alessandra Saraga, Nathan David Vanshelboym Rothschild, Mostafa A. Soliman, Audrey N. Kim, Isabella Midura, Rishita Tikkala, Lina Zhang, Shana Rakowsky, Loren Rabinowitz, Konstantinos Papamichael, Adam S. Cheifetz, Sarah Ballou

**Affiliations:** 1 Department of Medicine, Beth Israel Deaconess Medical Center, Boston, MA, United States; 2 Division of Gastroenterology, Beth Israel Deaconess Medical Center, Boston, MA, United States

**Keywords:** Crohn's disease, healthcare utilization, inflammatory bowel disease, treatment failure, ulcerative colitis

## Abstract

**Introduction:**

Patients with Inflammatory Bowel Disease (IBD) experience Irritable Bowel Syndrome (IBS)-like symptoms more frequently than the general population. The impact of IBS-like symptoms on the clinical course of IBD remains uncertain.

**Methods:**

In a prospective cohort of patients with quiescent IBD, as defined by endoscopic remission, IBS-like symptoms were measured using the IBS-Symptom Severity Scale (IBS-SSS) at the time of endoscopy to define IBD patients with no IBS-like symptoms (IBS-SSS <75), mild IBS-like symptoms (IBS-SSS 75-175), and moderate-severe IBS-like symptoms (IBS-SSS >175). The primary outcome was total IBD-related healthcare utilization (HCU), while secondary outcomes included time to unscheduled/acute HCU and treatment failure among patients on biologics.

**Results:**

Among 114 patients with quiescent IBD, 53 had no IBS-like symptoms, 43 had mild IBS-like symptoms, and 18 had moderate-severe IBS-like symptoms. The median follow-up time was 3.5 years and was comparable among groups (p=0.330). Patients with mild or moderate-severe IBS-like symptoms had a higher total number of HCU events than patients without IBS-like symptoms (median (IQR): 8 (6–13) vs. 12 (9–15) vs 7 (5–11), respectively, p=0.042). After adjusting for IBD type and biologic therapy at baseline multivariable Cox proportional hazards regression analysis identified mild (HR: 3.6; 95%CI): 1.1-11.7; p=0.029) and moderate/severe (HR: 6.2; 95%CI: 1.8-21.3, p=0.004) IBS-like symptoms associated with unscheduled/acute HCU. Treatment failure did not differ by presence of IBS-like symptoms.

**Discussion:**

IBS-like symptoms in IBD are associated with increased HCU but not treatment failure. These findings suggest that IBS-like symptom burden in IBD is clinically meaningful, may drive healthcare seeking, and warrants targeted treatment within IBD care models.

## Introduction

Inflammatory bowel disease (IBD) is a chronic, immune-mediated disease of the gastrointestinal tract comprised of two main subtypes, ulcerative colitis (UC) and Crohn’s disease (CD). Patients with IBD often continue to experience abdominal pain, altered bowel habits, and bloating despite disease remission, now termed IBD with Irritable Bowel Syndrome (IBS)-like symptoms (1). The mechanisms of this relationship have remained elusive, with possible post-inflammatory visceral hypersensitivity, microbiome changes, dysregulated gut-brain interactions, intestinal permeability, and/or immune activation at play (1). The presence of IBS-like symptoms in IBD is associated with worse quality of life and increased psychological distress (1–3), potentially on par to the impacts of active IBD (2). Disentangling these entities is crucial to prevent unnecessary escalation or discontinuation of therapies for patients with symptoms that do not represent IBD disease activity (4) and provide more targeted treatment.

Estimates of the prevalence of IBS-like symptoms in IBD have ranged widely due to difficulty measuring IBS in the IBD population and the changing definitions of IBS. A systematic review and meta-analysis of 27 observational studies found a pooled prevalence of IBS in IBD of 32.5% (5), substantially higher than the IBS rate of about 6% observed in the general population (6). However, the prevalence of IBS in IBD varied according to how remission was defined, with lower rates observed when endoscopic data was utilized (5). More recently, a prospective analysis utilizing concurrent IBS symptom and endoscopic evaluation found a much lower rate of IBS in IBD compared to prior studies (7.9% by Rome IV criteria and 15.2% by moderate-to-severe IBS-Symptom Severity Score), suggesting that when strict criteria are used, IBS-like symptoms in IBD may be less common than was previously thought (7).

Beyond prevalence estimates, the clinical implications of IBS-like symptoms in the course of IBD remain unclear. Only two studies from a cohort in the United Kingdom (UK) have explored this question, finding that IBD patients with IBS-like symptoms had greater HCU but did not have worse longitudinal disease outcomes over time (2, 8). These studies, however, had limited ability to distinguish IBS-like symptoms from IBD since they lacked endoscopic data and utilized a suboptimal fecal calprotectin (FCP) cutoff of 250 ug/g (9) to define quiescent IBD.

The aim of this study was therefore to utilize a cohort of patients with IBD and evaluation of IBS-like symptoms by concurrent symptom and endoscopic evaluation to determine the longitudinal impacts of IBS-like symptoms in IBD.

## Materials and methods

### Study design and population

This was a single-center study of a longitudinal retrospective follow-up of a prospectively recruited cohort at a tertiary care academic medical center (7). Adults aged 18 and older with established IBD were approached at the time of index colonoscopy (between 2020 and 2022) to complete study questionnaires including demographic and baseline clinical data. Participants completed the IBS-Severity Scoring System (IBS-SSS), a validated tool for studying IBS-like symptoms in IBD (10). Patients with quiescent IBD, defined as endoscopic remission (endoscopic Mayo score 0 or 1 for UC and no ulcerations for CD) at the index colonoscopy (11), were stratified into three groups: no IBS-like symptoms (IBS-SSS score <75), mild IBS-like symptoms (IBS-SSS score 75-175), and moderate-severe IBS-like symptoms (IBS-SSS score >175). Clinical data through 9/30/2025 were then manually extracted from the electronic medical record by two physicians ([SM], [AS]). A third physician ([AC]) was available for consultation to resolve questions or discrepancies as needed. Patients were excluded if they did not achieve a minimum follow-up time of 90 days after index colonoscopy.

### Study outcomes

The primary outcome was total IBD-related healthcare utilization (HCU), defined as the total number of IBD-related HCU events including IBD-related clinic visits, endoscopic and imaging evaluations, emergency department visits, hospitalizations, and surgeries until the end of follow up. IBD-related clinic visits were defined as visits with a gastrointestinal specialist regarding symptoms. This included both regular scheduled follow-ups and additional appointments due to symptom burden. All subsequent endoscopic evaluations and any imaging (including CT, MRI, CT enterography or MR enterography) obtained with the purpose of evaluating IBD disease activity within the study period were also included. Emergency department visits and hospitalizations were judged to be IBD-related if gastrointestinal symptoms were thought to be due to IBD disease activity. IBD-associated conditions such as venous thrombosis as well as complications of IBD medications were also included. The HCU could be physician or patient driven.

A secondary outcome was time to unscheduled/acute HCU defined as imaging evaluations, emergency department visits, hospitalizations, and surgeries. IBD-related clinic visits and endoscopic evaluation were excluded, as these are part of the standard of care for monitoring IBD. Another secondary outcome was time to treatment failure defined as drug discontinuation due to primary non-response, loss of response, or serious adverse event and was evaluated only among patients receiving biologics at baseline.

### Statistical analysis

Qualitative data are presented as counts and percentages, and quantitative data are presented as median (interquartile range [IQR]). Continuous and categorical variables were compared between groups using the Kruskal-Wallis test and the chi-square or Fisher’s exact test, as appropriate, respectively. A generalized linear model with a negative binomial distribution using log follow-up time as an offset was performed to model the number of HCU events based on IBS-like symptoms and provide adjusted incidence rate ratio (aIRR) adjusting for IBD type and biologic therapy at baseline.

A time-to-event survival analysis was performed for unscheduled/acute HCU and treatment failure using Kaplan-Meier curves. Patients were followed from the time of the index colonoscopy until HCU or treatment failure or the end of follow up. The proportional hazards assumption was assessed using log-minus-log survival plots. Multivariable Cox proportional hazard regression analyses were performed to determine the independent effect of IBS-like symptoms associated with unscheduled/acute HCU adjusting for IBD type and biologic therapy at baseline. All analyses were performed using SPSS version 29.0 (SPSS, Chicago, IL, USA) and GraphPad Prism version 5.03 for Windows (GraphPad Software, San Diego, CA, USA).

## Results

### Study population

The study population consisted of 114 patients with quiescent IBD [no IBS-like symptoms (n=53); mild IBS-like symptoms (n=43), moderate/severe IBS-like symptoms (n=18), **Table 1]**. The great majority of the patients [96/114 (84.2%)] had also histological remission. The median follow-up time was 3.5 (IQR: 3-4) years and was comparable between groups [3.6 (3.2-4.1) vs. 3.3 (2.9-3.9) vs. 3.7 (3.3-4), p=0.330]. The patients’ demographic and clinical characteristics were comparable among the three groups, although more patients with IBS-like symptoms had Crohn’s Disease (CD) compared to patients without IBS-like symptoms, and more patients with moderate/severe IBS-like symptoms were treated with biologics at the time of the index colonoscopy compared to those with mild or no IBS-like symptoms (**Table 1**).

**Table 1:** Demographics and clinical characteristics of the study population.

Characteristics of the patients	Total (n=114)	Mild IBS-like symptoms (n=43)	Moderate/severe IBS-like symptoms (n=18)	No IBS-like symptoms (n=53)	P-value
Male, n (%)	52 (46)	20 (47)	6 (33)	26 (49)	0.299
Age, median (IQR), years	52 (41–65)	50 (34–65)	50 (41–63)	53 (45–68)	0.278
IBD type, n (%)					0.044
UC	58 (51)	18 (42)	6 (33)	34 (64)	
CD	47 (41)	23 (54)	10 (56)	14 (26)	
IBDU	9 (8)	2 (4)	2 (11)	5 (10)	
UC extension, n (%)					0.553
E1 (proctitis)	5/58 (8)	2/18 (11)	0/6 (0)	3/34 (9)	
E2 (left-sided colitis)	19/58 (33)	5/18 (28)	2/6 (33)	12/34 (35)	
E3 (pancolitis)	34/58 (59)	11/18 (61)	4/6 (67)	19/34 (56)	
CD location, n (%)					0.099
L1 (ileitis)	11/47 (24)	7/23 (30)	4/10 (40)	0/14 (0)	
L2 (colitis)	10/47 (21)	6/23 (27)	0/10 (0)	4/14 (29)	
L3 (ileocolitis)	26/47 (55)	10/23 (43)	6/10 (60)	10/14 (71)	
CD behavior, n (%)					0.875
B1 (non-stricturing, non-penetrating)	23/47 (49)	10/23 (43)	6/10 (60)	7/14 (50)	
B2 (stricturing)	9/47 (19)	7/23 (30)	0/10 (0)	2/14 (14)	
B3 (penetrating)	15/47 (32)	6/23 (27)	4/10 (40)	5/14 (36)	
Prior intestinal resection for CD, n (%)	15/47 (32)	9/23 (39)	2/10 (20)	4/14 (29)	0.752
Biologic therapy at baseline*, n (%)	59 (52)	22 (51)	15 (83)	22 (42)	0.004
IMN (azathioprine, or methotrexate), n (%)	13 (11)	5 (12)	1 (6)	7 (13)	0.418
Corticosteroids, n (%)	1 (1)	0 (0)	0 (0)	1 (2)	0.340
Anxiety (PROMIS) mean (SD)	51.9 (8.9)	54.7 (7.3)	58.9 (8.2)	47.2 (7.7)	<0.001
Depression (PROMIS) mean (SD)	46.6 (8.2)	48.8 (8.6)	51.2 (8.7)	43.2 (6.3)	<0.001

^*^ at the time of the index colonoscopy; ^#^PROMIS scores are t-scores (population mean = 50, SD, 10).

IBS, irritable bowel syndrome; IQR, interquartile range; IBD, inflammatory bowel disease; CD, Crohn’s disease; UC, ulcerative colitis; IBDU, inflammatory bowel disease unclassified; IMM, immunomodulators: PROMIS, Patient-Reported Outcomes Measurement Information System; SD, standard deviation.

### Healthcare utilization

There was a difference in the total number of HCU events among patients with mild or moderate/severe IBS-like symptoms and without IBS-like symptoms [median (IQR): 8 (6–13) vs. 12 (9–15) vs 7 (5–11), respectively, p=0.042, **Figure 1]**. Similarly, patients with mild or moderate/severe IBS-like symptoms had a higher number of HCU events per patient-year compared to patients without IBS-like symptoms (391/132.3 vs. 223/62.9 vs 447/183.7, respectively). Additional information regarding total HCU events among groups is provided in **Table 2**. A negative binomial model showed a higher aIRR for HCU in patients with mild [1.214 (95%CI: 1.012-1.456), p=0.035] or moderate/severe IBS-like symptoms [1.393 (95%CI: 1.106-1.753), p=0.004] compared to patients without IBS-like symptoms.

**Figure 1:**
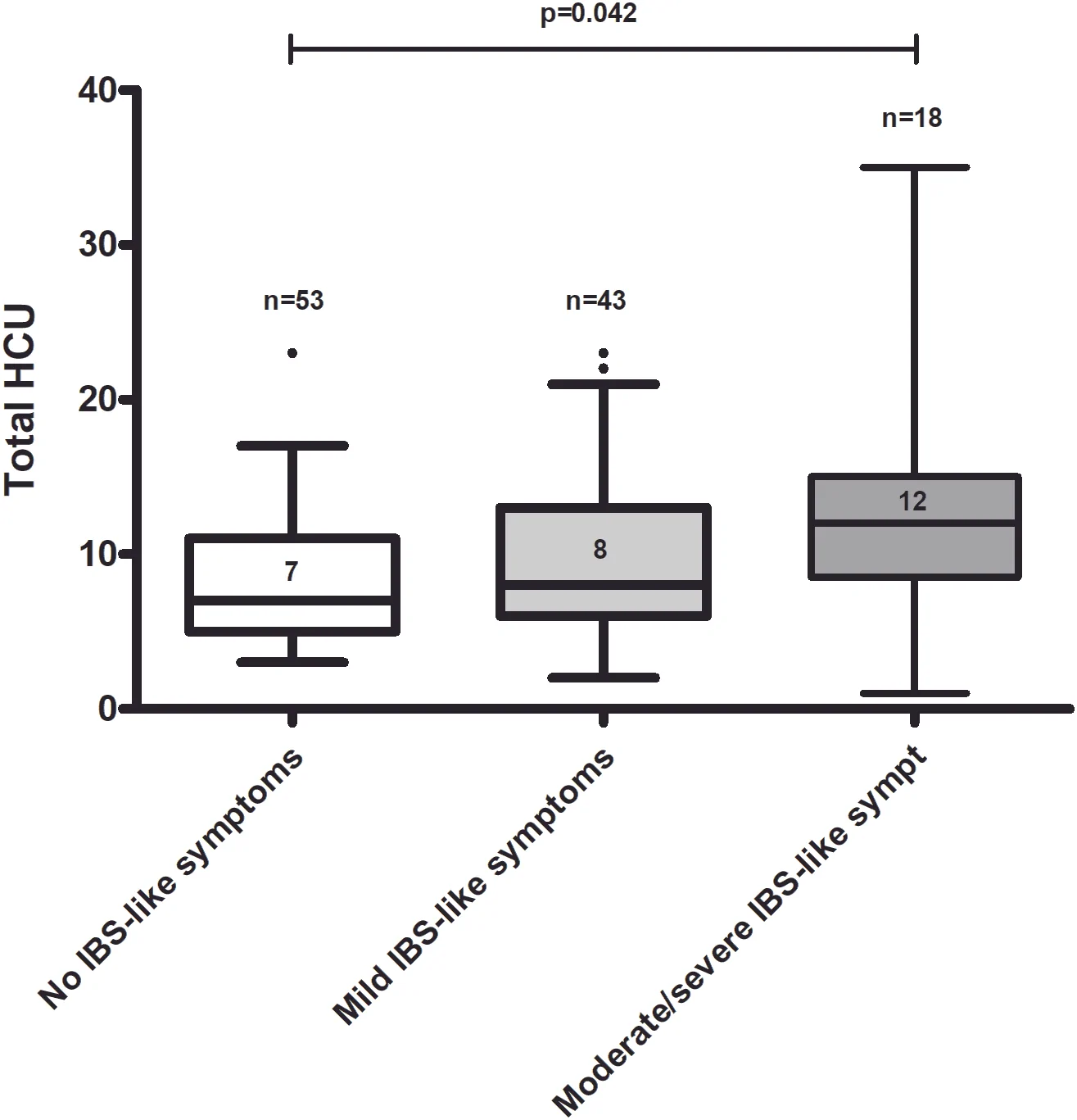
Total healthcare utilization (HCU) among patients with IBD with or without IBS-like symptoms. Box plots show the median (solid line within box), interquartile range (upper and lower box boundaries), 5-95% ranges (whiskers) and outliers (black dot).

**Table 2:** Total health care utilization event among groups.

Healthcare utilization	Mild IBS-like symptoms	Moderate/severe IBS-like symptoms	No IBS-like symptoms
Total HCU events, n	391	223	447
ED visits, n, (%)	0 (0)	4 (2)	1 (0)
Hospitalizations, n, (%)	2 (0)	1 (0)	5 (1)
Clinic visits, n, (%)	311 (80)	174 (78)	347 (78)
Endoscopies, n, (%)	56 (14)	27 (12)	89 (20)
Imaging, n, (%)	22 (6)	17 (8)	5 (1)
Surgery, n, (%)	0 (0)	0 (0)	0 (0)

IBS, irritable bowel syndrome; HCU, healthcare utilization; ED, emergency department.

Moreover, a time to event analysis showed that patients with IBS-like symptoms, either mild or moderate/severe, had a higher cumulative probability of unscheduled/acute HCU than patients without IBS-like symptoms (**Figure 2**). Of note, the curves rise almost entirely within the first year and then plateau. After adjusting for IBD type and biologic therapy at baseline multivariable Cox proportional hazards regression analysis identified mild (HR: 3.6; 95%CI): 1.1-11.7; p=0.029) and moderate/severe (HR: 6.2; 95%CI: 1.8-21.3, p=0.004) IBS-like symptoms associated with unscheduled/acute HCU. Given the small number of events, the HRs should be considered as exploratory.

**Figure 2:**
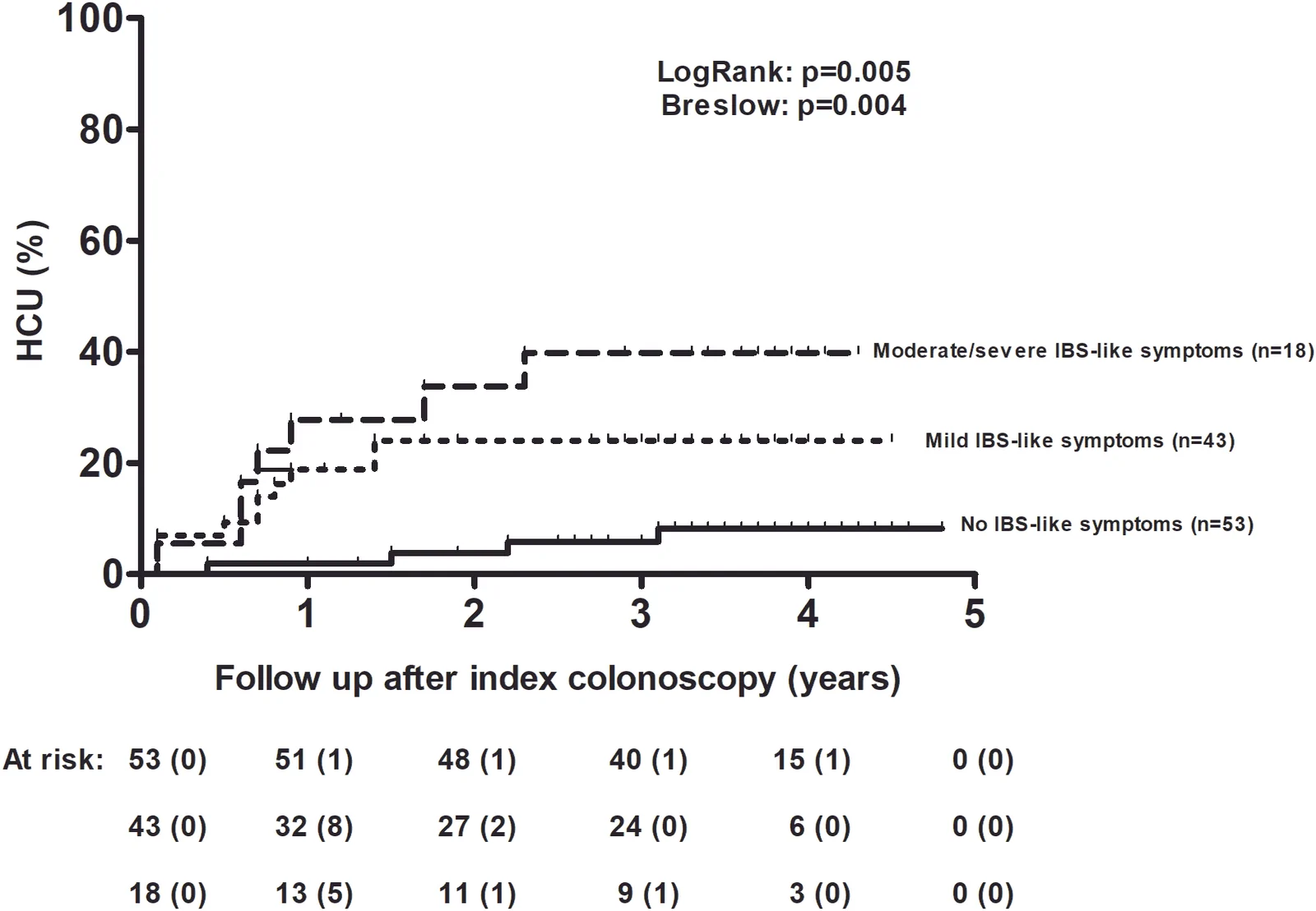
Time to first unscheduled/acute healthcare utilization (HCU) among IBD patients with or without IBS-like symptoms. Number of new events per year for each group are provided in parentheses. The mean survival time (95%CI) for IBD patients with mild or moderate/severe IBS-like symptoms compared to patients without IBS symptoms was 3.6 (3.1-4.1) vs. 3 (2.2-3.8) vs 4.6 (4.3-4.8), respectively.

### Treatment failure

There was no statistically significant difference in treatment failure among patients without IBS-like symptoms and with mild or moderate-severe IBS-like symptoms [2/22 (9.1%) vs. 4/15 (27%) vs. 2/22 (9.1%), respectively, p=0.156, linear-by-linear association). A time to event analysis showed that patients with IBS-like symptoms either mild or moderate-severe had a comparable cumulative probability of treatment failure compared to patients without IBS-like symptoms (Log Rank, p=0.589).

## Discussion

In this single-center cohort of patients with quiescent IBD, we demonstrated that IBS-like symptoms were independently associated with greater HCU over a median follow-up of 3.5 years. After adjusting for IBD type and biologic therapy, mild and moderate/severe IBS-like symptoms were associated with unscheduled/acute HCU. As shown in **Table 2**, the excess HCU was driven mainly by imaging rather than endoscopies, which were proportionally higher in the group without IBS symptoms, or hospitalizations and emergency visits, which were few. This along with the possibility of surveillance bias and the fact that patients with IBS symptoms had more anxiety and depression may explain the higher rates of HCU in this population compared to patients without IBS symptoms. These findings build upon prior work by leveraging concurrent endoscopic and symptom evaluation to more rigorously characterize the longitudinal impacts of IBS-like symptoms in IBD. Our finding that IBS-like symptoms are associated with increased HCU is consistent with two prior studies from a UK cohort which similarly identified greater healthcare use among IBD patients with IBS-like symptoms (2, 8).

Our study also showed that treatment failure among patients on biologic therapy did not differ significantly between groups, although it was numerically higher in patients with moderate/severe IBS-like symptoms compared to patients with mild or no IBS-like symptoms. Similarly, Gracie et al. (2) and Fairbrass et al. (8) did not find an association between IBS symptoms and longitudinal disease activity, including need for glucocorticoid, escalation of medical therapy due to uncontrolled disease activity, hospitalization, or intestinal resection. It is important to note that Gracie et al. (2) and Fairbrass et al. (8) did not incorporate endoscopic data to define quiescent IBD and instead utilized FCP cutoff of 250 μg/g, which has been shown to be a suboptimal threshold to exclude ongoing IBD activity.

This study has several notable strengths. These included the use of concurrent endoscopic and symptom assessment allowing for more rigorous stratification of patients with versus without IBS-like symptoms compared to prior studies, the longitudinal long-term follow-up of 3.5 years providing sufficient time to observe clinically meaningful outcomes, and the fact that quiescent IBD was defined based on endoscopic remission at the index colonoscopy.

Several limitations should also be noted. Though our study includes more rigorous evaluation of IBS in IBD compared to prior work, defining IBS in this population remains challenging. Other etiologies such as bile acid diarrhea, small intestine bacterial overgrowth, or pelvic floor dysfunction, may cause ongoing symptoms and independently impact IBD disease course. In addition, symptoms could be caused by strictures or residual microscopic inflammation, since proximal small bowel activity could not be fully excluded. Fecal calprotectin, C-reactive protein as well as radiological or sonographic data regarding the definition of quiescent disease were available only for a small portion of the study population. Additionally, the IBS-SSS was also administered only at baseline, although IBS-type symptoms may fluctuate over time. The modest sample size made subgroup analyses between patients with CD vs UC, male vs female or different age groups not feasible. Moreover, our population at a tertiary care center may not be representative of the general IBD population. Furthermore, unmeasured confounding may persist despite well-balanced baseline variables and the use of multivariable analyses, including the potential impact of the COVID-19 pandemic on early follow-up utilization as the index colonoscopies were done between 2020 and 2022.

In conclusion, we found that IBS-like symptoms in patients with endoscopically quiescent IBD were associated with increased HCU, although not with biologic treatment failure. These findings suggest that IBS-like symptom burden in quiescent IBD is clinically meaningful, may drive healthcare-seeking, and warrants targeted recognition and treatment within IBD care models.

## Data Availability

The raw data supporting the conclusions of this article will be made available by the authors, without undue reservation.

## References

[1] Ma C Ford AC Hashash JG Barbara G Bernstein CN Brenner DM . Recommendations for the evaluation and management of inflammatory bowel disease with irritable bowel syndrome–like symptoms: A joint Rome Foundation and International Organization for the Study of IBD (IOIBD) consensus. Gastroenterology. (2026) 171, 504-20. doi: 10.1053/j.gastro.2026.04.008 42066865

[2] Gracie DJ Hamlin JP Ford AC . Longitudinal impact of IBS-type symptoms on disease activity, healthcare utilization, psychological health, and quality of life in inflammatory bowel disease. Am J Gastroenterol. (2018) 113:702–12. doi: 10.1038/s41395-018-0021-z 29453384

[3] Ozer M Bengi G Colak R Cengiz O Akpinar H . Prevalence of irritable bowel syndrome-like symptoms using Rome IV criteria in patients with inactive inflammatory bowel disease and relation with quality of life. Med (Baltimore). (2020) 99:e20067. doi: 10.1097/MD.0000000000020067 32384473 PMC7220554

[4] Elias ED Bernstein CN Singh H Oketola A Krishnan S Targownik LE . Dose augmentation of tumor necrosis factor inhibitors is frequently performed in persons with inflammatory bowel disease in the absence of objective evidence of active inflammation. J Clin Gastroenterol. (2021) 55:602. doi: 10.1097/MCG.0000000000001391 32694264

[5] Fairbrass KM Costantino SJ Gracie DJ Ford AC . Prevalence of irritable bowel syndrome-type symptoms in patients with inflammatory bowel disease in remission: A systematic review and meta-analysis. Lancet Gastroenterol Hepatol. (2020) 5:1053–62. doi: 10.1016/S2468-1253(20)30300-9 33010814

[6] Almario CV Sharabi E Chey WD Lauzon M Higgins CS Spiegel BMR . Prevalence and burden of illness of Rome IV irritable bowel syndrome in the United States: Results from a nationwide cross-sectional study. Gastroenterology. (2023) 165:1475–87. doi: 10.1053/j.gastro.2023.08.010 37595647

[7] Burdine LK Rakowsky S Grossberg L Rabinowitz L Cheifetz AS Ballou S . Irritable bowel syndrome/inflammatory bowel disease overlap: Less common than we think. Gastro Hep Adv. (2024) 3:1135–7. doi: 10.1016/j.gastha.2024.08.005 39555317 PMC11564024

[8] Fairbrass KM Hamlin PJ Gracie DJ Ford AC . Natural history and impact of irritable bowel syndrome-type symptoms in inflammatory bowel disease during 6 years of longitudinal follow-up. Aliment Pharmacol Ther. (2022) 56:1264–73. doi: 10.1111/apt.17193 35995729

[9] Dajti E Frazzoni L Iascone V Secco M Vestito A Fuccio L . Systematic review with meta-analysis: Diagnostic performance of faecal calprotectin in distinguishing inflammatory bowel disease from irritable bowel syndrome in adults. Aliment Pharmacol Ther. (2023) 58:1120–31. doi: 10.1111/apt.17754 37823411

[10] Francis CY Morris J Whorwell PJ . The irritable bowel severity scoring system: A simple method of monitoring irritable bowel syndrome and its progress. Aliment Pharmacol Ther. (1997) 11:395–402. doi: 10.1046/j.1365-2036.1997.142318000.x 9146781

[11] Deyhim T Saraga A Gade A Geeganage G Soliman MA Zullow S . Lower drug clearance of adalimumab is associated with proactive therapeutic drug monitoring and mucosal healing in patients with inflammatory bowel disease. Eur J Gastroenterol Hepatol. (2026) 38:1030–5. doi: 10.1097/MEG.0000000000003183 41870904

